# Synthesis, crystal structure and Hirshfeld surface analysis of the hybrid salt bis­(2-methyl­imidazo[1,5-*a*]pyridin-2-ium) tetra­chlorido­manganate(II)

**DOI:** 10.1107/S2056989023002761

**Published:** 2023-03-28

**Authors:** Olga Yu. Vassilyeva, Elena A. Buvaylo, Vladimir N. Kokozay, Brian W. Skelton

**Affiliations:** a Department of Chemistry, Taras Shevchenko National University of Kyiv, 64/13 Volodymyrska Street, Kyiv 01601, Ukraine; b School of Molecular Sciences, M310, University of Western Australia, Perth, WA 6009, Australia; Universidad Nacional Autónoma de México, México

**Keywords:** crystal structure, organic–inorganic hybrid, pseudo-layered structure, 2-pyridine­carbaldehyde

## Abstract

The 0-D hybrid salt bis­(2-methyl­imidazo[1,5-*a*]pyridin-2-ium) tetra­chlorido­manganate(II), (C_8_H_9_N_2_)_2_[MnCl_4_] or [*L*]_2_[MnCl_4_], consists of discrete *L* ^+^ cations and tetra­chlorido­manganate(II) anions. The fused heterocyclic rings in the two crystallographically non-equivalent monovalent organic cations are almost coplanar; the bond lengths are as expected. The tetra­hedral MnCl_4_^2–^ dianion is slightly distorted with the Mn—Cl bond lengths varying from 2.3577 (7) to 2.3777 (7) Å and the Cl—Mn—Cl angles falling in the range 105.81 (3)–115.23 (3)°. In the crystal, the compound demonstrates a pseudo-layered arrangement of separate organic and inorganic sheets alternating parallel to the *bc* plane. In the organic layer, pairs of centrosymmetrically related *trans*-oriented *L* ^+^ cations are π-stacked. Neighboring MnCl_4_^2–^ dianions in the inorganic sheet show no connectivity, with the minimal Mn⋯Mn distance exceeding 7 Å. The Hirshfeld surface analysis revealed the prevalence of the non-conventional C—H⋯Cl—Mn hydrogen bonding in the crystal packing.

## Abstract 2

The 0-D hybrid salt (C_8_H_9_N_2_)_2_[MnCl_4_] with a pseudo-layered arrangement of the organic and inorganic sheets is isomorphous with the Zn and Cd analogues. According to the Hirshfeld surface analysis, non-conventional C—H⋯Cl—Mn hydrogen bonding is predominant in the crystal packing.

## 1. Chemical context

Hybrid metal halides combining organic cations and inorganic anions are the focus of research attention as novel light-emitting materials because their photoluminescence properties are conveniently tunable by engineering their organic and inorganic components (Saparov & Mitzi, 2016 ▸). These mat­erials have potential uses in light-emitting diodes (LEDs), solar cells, and photodetectors as well as in laser technology (Li *et al.*, 2021 ▸). The Pb element in this family, however, prevents these materials from being used in commercial settings (Gan *et al.*, 2021 ▸). Therefore, the development of lead-free hybrid metal halides is of particular inter­est. Environmentally safe organic–inorganic manganese(II) halides have been shown to exhibit potent luminescence arising from *d*–*d* transitions, making them promising for use in X-ray scintillators, sensors, and optical devices (Kumar Das *et al.*, 2022 ▸). 
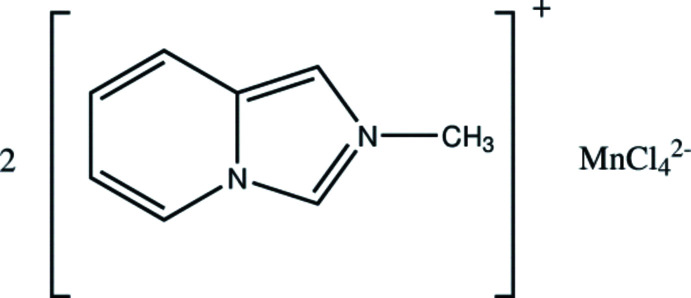


The title compound [*L*]_2_[MnCl_4_], (**I**), was synthesized in the course of our study on organic–inorganic hybrid metal halides of transition and main-group metal atoms counterbalanced with imidazo[1,5-a]pyridinium-based cations (Vassilyeva *et al.*, 2020 ▸, 2021 ▸, 2023 ▸). The monovalent 2-methyl-imidazo[1,5-*a*]pyridinium cation *L* ^+^ resulted from the oxidative condens­ation–cyclization between formaldehyde, methyl­amine hydro­chloride and 2-pyridine­carbaldehyde. The reaction of the preformed heterocyclic cation and metal halides yielded hybrid compounds [*L*]_*n*_[PbCl_3_]_*n*_ (TURJUO; Vassilyeva *et al.*, 2020 ▸), [L]_2_[ZnCl_4_] (GOTHAB01; Vassilyeva *et al.*, 2020 ▸), [*L*]_2_[CdCl_4_] (GOTJAD01; Vassilyeva *et al.*, 2021 ▸), and [*L*]_2_[SnCl_6_] (GIBFAC; Vassilyeva *et al.*, 2023 ▸). The photophysical properties of the organic–inorganic 1-D perovskite [*L*]_*n*_[PbCl_3_]_*n*_ and 0-D pseudo-layered hybrid [*L*]_2_[ZnCl_4_] were presumed to originate from the synergistic effects of the electronic structure of the cation and the solid-state architectures. Hybrid compound **I**, isomorphic with the Zn and Cd analogues GOTHAB01 and GOTJAD01, appeared non-emissive. Herein, the synthesis, structure, IR spectroscopic characterization, and the results of the Hirshfeld surface (HS) analysis of **I** are reported.

## 2. Structural commentary

**Figure 1:**
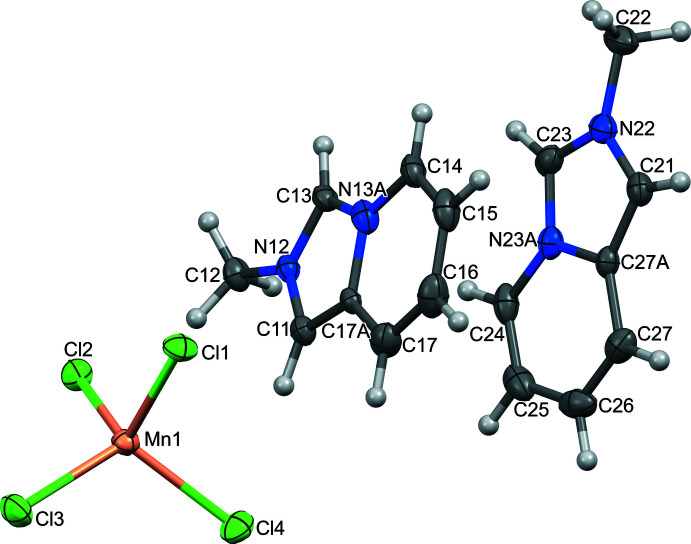
Mol­ecular structure and atom labeling of [*L*]_2_[MnCl_4_] (**I**), with 50% probability displacement ellipsoids.

The organic–inorganic hybrid salt **I** crystallizes in the triclinic space group *P* 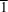 and is isomorphous with the [*L*]_2_[ZnCl_4_] (GOTHAB; Vassilyeva *et al.*, 2020 ▸) and [*L*]_2_[CdCl_4_] (GOTJAD; Vassilyeva *et al.*, 2021 ▸) analogues as well as the sister mixed-halide Zn^II^ and Cd^II^ tetra­halometalates with the *L* ^+^ cation involving partial substitution of bromide by chloride and chloride by iodide ions (NOTZAA01, NOVSEZ01 and NOVSOJ01; Vassilyeva *et al.*, 2022 ▸). Compound **I** is composed of discrete *L* ^+^ cations and tetra­hedral MnCl_4_^2–^ anions (Fig. 1 ▸). In the asymmetric unit, there are two crystallographically non-equivalent cations (N22, N23*A* and N12, N13*A*) with similar structural configurations, which are very close to those of the isomorphous hybrid salts. In the fused cores, the imidazolium rings show C—N/C bond lengths in the range 1.332 (3)–1.405 (3) Å; the pyridinium rings have normal bond distances; the nitro­gen atoms are planar, with a total sum of three angles of 360°. The five- and six-membered rings in the cations are almost coplanar, showing dihedral angles between them of less than 2° [0.61 (N22, N23*A*) and 1.46° (N12, N13*A*)].

The geometry of the slightly distorted tetra­hedral MnCl_4_^2–^ anion with Mn—Cl distances varying from 2.3577 (7) to 2.3777 (7) Å and the bond angles falling in the range 105.81 (3)–115.23 (3)° (Table 1 ▸) is typical for this coordinatively rigid anion.

## 3. Supra­molecular features

**Figure 2:**
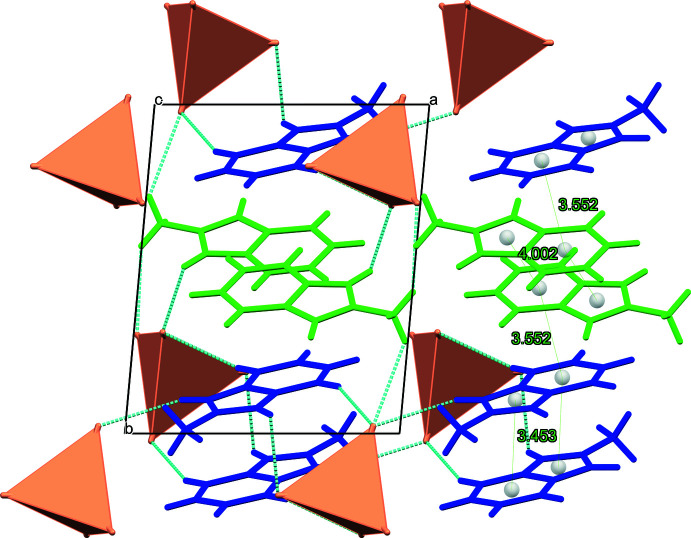
Fragment of the crystal packing of **I** viewed along the *c* axis with the non-equivalent *L*1^+^ and *L*2^+^ cations shown in blue and green, [MnCl_4_]^2–^ anions are presented in a polyhedral form, and C—H⋯Cl—Mn hydrogen bonds are shown in blue.

In the crystal of **I**, there is a pseudo-layered arrangement of the organic and inorganic sheets alternating parallel to the *bc* plane (Fig. 2 ▸). The *a*-axis length [9.4042 (6) Å] corresponds to the distance between consecutive inorganic planes. Pairs of inversion-related cations in the organic layer are stacked with varying levels of offset, showing the ring-centroid distances of 3.453 (1) (N22, N23*A*), 3.552 (2) and 4.002 (1) Å (N12, N13*A*) with the corresponding inter­planar distances being 3.263 (1), 3.526 (1) and 2.401 (1) Å, respectively. In the inorganic layer, the tetra­chlorido­manganate(II) anions are loosely packed with the shortest Mn⋯Mn separations being about 7.098 Å. The closest Cl⋯Cl distance of 4.649 Å is significantly larger than the double value of the Shannon ionic radii of chloride anion [2*r*(Cl^−^) = 2 × 1.81 = 3.62 Å], making magnetic inter­actions between the metal ions barely possible. Additional structure consolidation is provided by numerous C—H⋯Cl—Mn hydrogen bonds between organic and inorganic sublattices (Fig. 2 ▸, Table 2 ▸) at the H⋯Cl distances below the van der Waals contact limit of 2.85 Å (Mantina *et al.*, 2009 ▸).

## 4. Hirshfeld surface analysis

**Figure 3:**
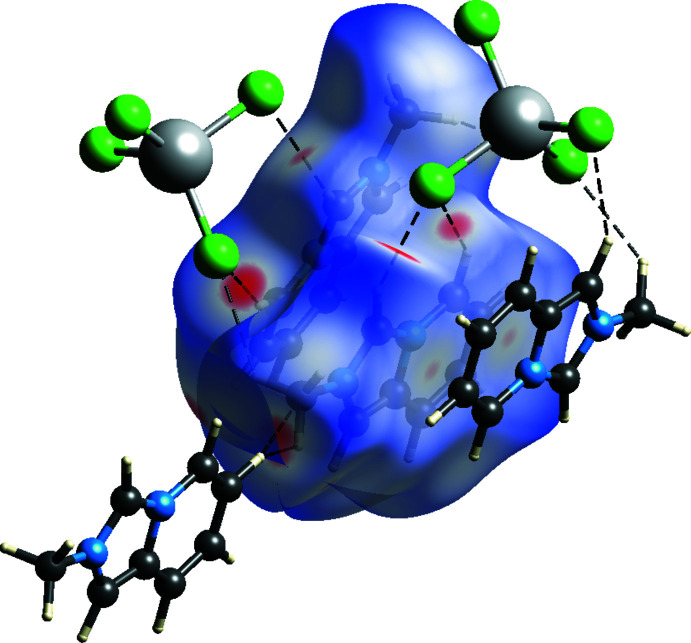
HS mapped over *d*_norm_ for the *L* ^+^ cations in [*L*]_2_[MnCl_4_] (**I**).

**Figure 4:**
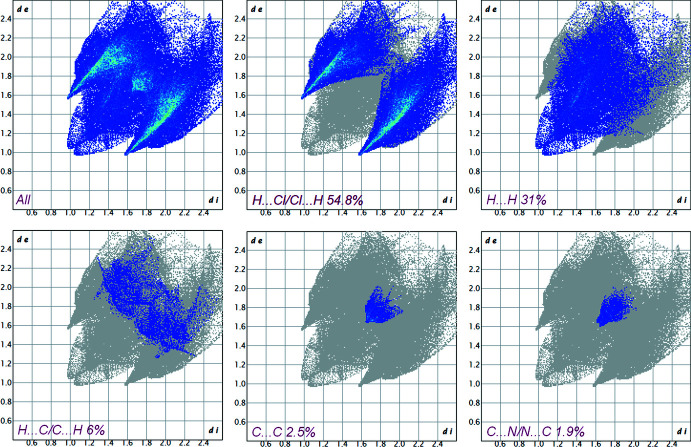
Selected two-dimensional fingerprint plots of compound **I** where *d*_i_ and *d*_e_ represent the distances from the HS to the closest inter­nal and external atoms.

The Hirshfeld surface mapped over *d*_norm_ and fingerprint plots for **I** were generated using *CrystalExplorer* (Version 21.5; Spackman *et al.*, 2021 ▸). The red spots on the Hirshfeld surface indicate close hydrogen-bond donor and acceptor contacts, while the white and blue areas represent van der Waals and longer contacts, respectively. The bright-red spots are found near chlorine atoms involved in C—H⋯Cl hydrogen-bonding inter­actions between organic cations and MnCl_4_^2–^ anions (Fig. 3 ▸). In the fingerprint plots (Fig. 4 ▸), those are associated with sharp spikes of 54.8% of the surface area. The next highest contributions to the surface contacts come from the H⋯H (31%), H⋯C (6%) and C⋯C (2.5%) inter­actions, whereas other *X*_i_⋯*X*_d_ contacts (*X* = H, N, C, Mn) cover less than 6% (Fig. 4 ▸). These numbers show that non-conventional hydrogen bonding predominates in the crystal packing of **I**, but that C–H⋯π and π–π inter­actions also make an appreciable contribution.

## 5. Database survey

Compound **I** is a new member of the family of salts with imidazo[1,5-*a*]pyridinium-based cations. More than 50 structures of the compounds including such cations are found in the Cambridge Structural Database (CSD, Version 5.42; Groom *et al.*, 2016 ▸) with 24 halometalates (*M* = Mn, Co, Fe, Ni, Cu, Zn, Cd, Pb and Sn) contributed by our research team. Another large group comprises organic salts with substituted *L* ^+^ cations and inorganic anions such as perchlorate or hexa­fluoro­phosphate. NAKNET (Mishra *et al.*, 2005 ▸) and DIWYEP (Kriechbaum *et al.*, 2014 ▸) with bulky methyl­phenyl and di­methyl­phenyl substituents, respectively, instead of the methyl group in *L* ^+^ are close analogues. A limited amount of the main-group metal halides with imidazo[1,5-*a*]pyridinium-based cations are known. The proligand bearing a 6-methyl­pyridin-2-yl substituent in place of the methyl group in *L* ^+^ (SOHPUC; Samanta *et al.*, 2014 ▸) was reported to stabilize both Au^I^ and Au^III^ ions, enabling the mixed-valence hybrid salt with [AuCl_2_]^−^ and [AuCl_4_]^−^ anions (SUWVIR; Nandy *et al.*, 2016 ▸). In the reaction with mercury(II) acetate, a similar ligand that lacked a methyl group, produced an Hg^II^–*N*-heterocyclic carbene complex of virtually linear geometry [C_carbene_—Hg—C_carbene_ = 176.56 (17)°] around the Hg center (IVOWEW; Samanta *et al.*, 2011 ▸).

The ubiquitous tetra­chloride anion is found in more than 200 structures stored in the CSD. The average Mn—Cl distance of 2.37 Å in **I** is comparable to those found in the database for other salts containing isolated MnCl_4_^2–^ tetra­hedral anions (the range of average Mn—Cl distances for this anion is 2.27–2.42 Å).

## 6. Synthesis and crystallization

Synthesis of [*L*]_2_[MnCl_4_], (**I**). Solid CH_3_NH_2_·HCl (0.27 g, 4 mmol) was added to the warm formaldehyde solution prepared by dissolving paraform (0.13 g, 4.5 mmol) in boiling deionized water (10 ml) in a 50 ml conical flask. The solution was stirred vigorously for 1 h at room temperature (r.t.), filtered and left open overnight. On the next day, 2-pyridine­carbaldehyde (0.19 ml, 2 mmol) was added to the flask, followed by Mn(OAc)_2_·4H_2_O (0.49 g, 2 mmol) dissolved in 5 ml of water, and the solution was magnetically stirred at r.t. for 30 min, then filtered and allowed to evaporate. Very light-brown needles of **I** suitable for X-ray crystallography formed within two days in the brown solution. The crystals were filtered off, washed with diethyl ether and dried in air. Yield: 67% (based on Mn). FT–IR (ν, cm^−1^): 3430*br*, 3122*s*, 3094*s*, 3050*s*, 3014, 2954, 2914, 2826, 1654, 1566, 1544, 1454, 1374, 1352, 1328, 1258, 1222, 1148*s*, 1130, 1038, 920, 800*s*, 764, 742, 624*s*, 498, 468, 434. Elemental analysis calculated for C_16_H_18_N_4_MnCl_4_ (463.08): C 41.50; H 3.92; N 12.10%. Found: C 41.62; H 3.93; N 12.04%.

**Figure 5:**
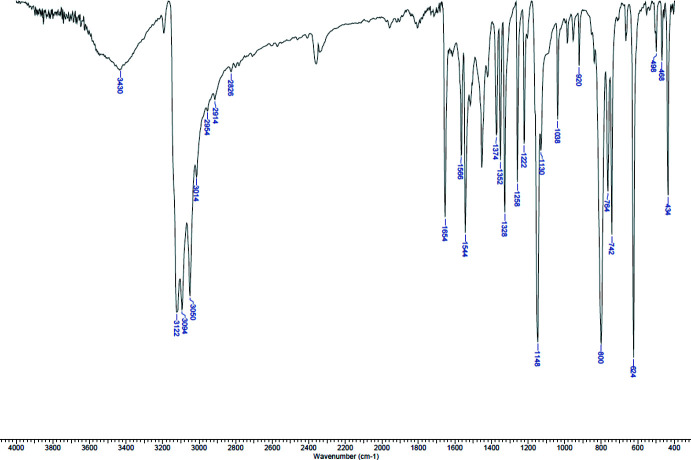
FT–IR spectrum of [*L*]_2_[MnCl_4_] (**I**) in KBr in the 4000–400 cm^−1^ range.

The FT–IR spectrum of **I** in KBr measured in the 4000–400 cm^−1^ range (Fig. 5 ▸) has a distinctive pattern characteristic of the imidazo[1,5-*a*]pyridinium-based skeleton (Vassilyeva *et al.*, 2020 ▸, 2021 ▸): the very strong sharp peaks are due to aromatic C—H stretching (3122–3050 cm^−1^), the medium intensity bands at 1654 and 1544 cm^−1^ are associated with heterocyclic ring vibrations, an intense absorption at 1148 cm^−1^ is ascribed to ν(N–C_CH3_) vibration and there is a prominent set of three peaks in the out-of-plane C—H-bending region (800, 742 and 624 cm^−1^).

## 7. Refinement

Crystal data, data collection and structure refinement details are summarized in Table 3 ▸. All hydrogen atoms were included in calculated positions and refined using a riding model with isotropic displacement parameters based on those of the parent atom (C—H = 0.95 Å, *U*_iso_(H) = 1.2*U*_eq_(C) for CH, C—H = 0.98 Å, *U*_iso_(H) = 1.5*U*_eq_(C) for CH_3_). Anisotropic displacement parameters were employed for the non-hydrogen atoms.

## Supplementary Material

Crystal structure: contains datablock(s) I, global. DOI: 10.1107/S2056989023002761/jq2027sup1.cif

Structure factors: contains datablock(s) I. DOI: 10.1107/S2056989023002761/jq2027Isup2.hkl

CCDC reference: 1959314

Additional supporting information:  crystallographic information; 3D view; checkCIF report

## Figures and Tables

**Table 1:** Selected geometric parameters (Å, °)

Mn1—Cl3	2.3577 (7)	Mn1—Cl1	2.3725 (7)
Mn1—Cl4	2.3674 (7)	Mn1—Cl2	2.3777 (7)
			
Cl3—Mn1—Cl4	115.23 (3)	Cl3—Mn1—Cl2	110.21 (3)
Cl3—Mn1—Cl1	106.36 (3)	Cl4—Mn1—Cl2	107.51 (3)
Cl4—Mn1—Cl1	105.81 (3)	Cl1—Mn1—Cl2	111.72 (2)

**Table 2:** Hydrogen-bond geometry (Å, °)

*D*—H⋯*A*	*D*—H	H⋯*A*	*D*⋯*A*	*D*—H⋯*A*
C11—H11⋯Cl4	0.95	2.76	3.592 (2)	146
C13—H13⋯Cl1^i^	0.95	2.66	3.422 (2)	138
C14—H14⋯Cl1^i^	0.95	2.79	3.549 (3)	137
C21—H21⋯Cl3^ii^	0.95	2.80	3.632 (2)	147
C22—H22*C*⋯Cl2^i^	0.98	2.82	3.742 (3)	156
C23—H23⋯Cl1^iii^	0.95	2.81	3.440 (3)	125
C24—H24⋯Cl3^iii^	0.95	2.69	3.573 (3)	154
C27—H27⋯Cl4^iv^	0.95	2.77	3.600 (3)	146

Symmetry codes: (i) 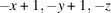 ; (ii) 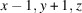 ; (iii) 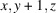 ; (iv) 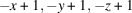 .

**Table 3:** Experimental details

Crystal data	
Chemical formula	(C_8_H_9_N_2_)_2_[MnCl_4_]
*M*_r_	463.08
Crystal system, space group	Triclinic, *P* 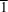
Temperature (K)	100
*a*, *b*, *c* (Å)	9.4042 (6), 10.7074 (6), 10.7401 (6)
α, β, γ (°)	99.211 (5), 110.852 (6), 91.515 (5)
*V* (Å^3^)	993.52 (11)
*Z*	2
Radiation type	Mo *K*α
μ (mm^−1^)	1.21
Crystal size (mm)	0.43 × 0.16 × 0.1

Data collection	
Diffractometer	Oxford Diffraction Xcalibur diffractometer
Absorption correction	Analytical (*CrysAlis PRO*; Rigaku OD, 2016 ▸)
*T*_min_, *T*_max_	0.706, 0.908
No. of measured, independent and observed [*I* > 2σ(*I*)] reflections	11038, 6374, 5151
*R*_int_	0.023
(sin θ/λ)_max_ (Å^−1^)	0.748

Refinement	
*R*[*F* ^2^ > 2σ(*F* ^2^)], *wR*(*F* ^2^), *S*	0.047, 0.126, 1.03
No. of reflections	6374
No. of parameters	228
H-atom treatment	H-atom parameters constrained
Δρ_max_, Δρ_min_ (e Å^−3^)	1.32, −0.65

Computer programs: *CrysAlis PRO* (Rigaku OD, 2016 ▸), *SHELXT* (Sheldrick, 2015*a* ▸), *SHELXL2017* (Sheldrick, 2015*b* ▸), *Mercury* (Macrae *et al.*, 2020 ▸), and *WinGX* publication routines (Farrugia, 2012 ▸).

## supplementary crystallographic information

### Crystal data

**Table:**

(C_8_H_9_N_2_)_2_[MnCl_4_]	*Z* = 2
*M**_r_* = 463.08	*F*(000) = 470
Triclinic, *P*1	*D*_x_ = 1.548 Mg m^−^^3^
Hall symbol: -P 1	Mo *K*α radiation, λ = 0.71073 Å
*a* = 9.4042 (6) Å	Cell parameters from 3962 reflections
*b* = 10.7074 (6) Å	θ = 2.1–31.2°
*c* = 10.7401 (6) Å	µ = 1.21 mm^−^^1^
α = 99.211 (5)°	*T* = 100 K
β = 110.852 (6)°	Needle, light brown
γ = 91.515 (5)°	0.43 × 0.16 × 0.1 mm
*V* = 993.52 (11) Å^3^	

### Data collection

**Table:**

Oxford Diffraction Xcalibur diffractometer	6374 independent reflections
Radiation source: Enhance (Mo) X-ray Source	5151 reflections with *I* > 2σ(*I*)
Graphite monochromator	*R*_int_ = 0.023
Detector resolution: 16.0009 pixels mm^-1^	θ_max_ = 32.1°, θ_min_ = 2.1°
ω scans	*h* = −13→13
Absorption correction: analytical (CrysAlis Pro; Rigaku OD, 2016)	*k* = −15→13
*T*_min_ = 0.706, *T*_max_ = 0.908	*l* = −11→15
11038 measured reflections	

### Refinement

**Table:**

Refinement on *F*^2^	0 restraints
Least-squares matrix: full	Hydrogen site location: inferred from neighbouring sites
*R*[*F*^2^ > 2σ(*F*^2^)] = 0.047	H-atom parameters constrained
*wR*(*F*^2^) = 0.126	*w* = 1/[σ^2^(*F*_o_^2^) + (0.0564*P*)^2^ + 0.8647*P*] where *P* = (*F*_o_^2^ + 2*F*_c_^2^)/3
*S* = 1.03	(Δ/σ)_max_ < 0.001
6374 reflections	Δρ_max_ = 1.32 e Å^−^^3^
228 parameters	Δρ_min_ = −0.65 e Å^−^^3^

### Special details

**Table:**

Geometry. All esds (except the esd in the dihedral angle between two l.s. planes) are estimated using the full covariance matrix. The cell esds are taken into account individually in the estimation of esds in distances, angles and torsion angles; correlations between esds in cell parameters are only used when they are defined by crystal symmetry. An approximate (isotropic) treatment of cell esds is used for estimating esds involving l.s. planes.
Refinement. One reflection which was considered to be obscured by the beam stop was omitted from the refinement.

### Fractional atomic coordinates and isotropic or equivalent isotropic displacement parameters (Å^2^)

**Table:**

	*x*	*y*	*z*	*U*_iso_*/*U*_eq_	
C11	0.7645 (3)	0.5468 (2)	0.2669 (2)	0.0213 (4)	
H11	0.838597	0.50728	0.330658	0.026*	
N12	0.7899 (2)	0.61515 (18)	0.18183 (18)	0.0182 (3)	
C12	0.9382 (3)	0.6391 (2)	0.1700 (3)	0.0256 (5)	
H12A	0.989164	0.719825	0.228348	0.038*	
H12B	0.922869	0.643492	0.075695	0.038*	
H12C	1.001665	0.570072	0.198003	0.038*	
C13	0.6572 (2)	0.6572 (2)	0.1041 (2)	0.0204 (4)	
H13	0.645655	0.70732	0.036315	0.024*	
N13A	0.5446 (2)	0.6135 (2)	0.1425 (2)	0.0266 (4)	
C14	0.3856 (3)	0.6248 (2)	0.1000 (3)	0.0253 (5)	
H14	0.336093	0.668856	0.02843	0.03*	
C15	0.3062 (3)	0.5714 (3)	0.1637 (3)	0.0315 (6)	
H15	0.199452	0.578985	0.136942	0.038*	
C16	0.3786 (3)	0.5039 (3)	0.2702 (3)	0.0352 (6)	
H16	0.319686	0.468677	0.314078	0.042*	
C17	0.5293 (3)	0.4893 (2)	0.3098 (3)	0.0303 (5)	
H17	0.576756	0.442786	0.37962	0.036*	
C17A	0.6142 (2)	0.54440 (19)	0.2455 (2)	0.0153 (4)	
C21	0.2932 (3)	0.8976 (2)	0.3195 (2)	0.0250 (5)	
H21	0.196989	0.897717	0.329702	0.03*	
N22	0.3271 (3)	0.9359 (2)	0.2170 (2)	0.0267 (4)	
C22	0.2201 (4)	0.9879 (3)	0.1047 (3)	0.0370 (6)	
H22A	0.275187	1.01841	0.051736	0.056*	
H22B	0.173674	1.058598	0.14116	0.056*	
H22C	0.139884	0.921389	0.046435	0.056*	
C23	0.4729 (3)	0.9225 (2)	0.2345 (2)	0.0260 (5)	
H23	0.523166	0.942318	0.176507	0.031*	
N23A	0.5364 (2)	0.87586 (19)	0.3492 (2)	0.0232 (4)	
C24	0.6858 (3)	0.8453 (2)	0.4119 (3)	0.0289 (5)	
H24	0.761335	0.85674	0.374017	0.035*	
C25	0.7194 (3)	0.7991 (3)	0.5275 (3)	0.0337 (6)	
H25	0.819593	0.775808	0.570203	0.04*	
C26	0.6093 (3)	0.7844 (2)	0.5870 (3)	0.0334 (6)	
H26	0.63787	0.75435	0.670355	0.04*	
C27	0.4650 (3)	0.8124 (2)	0.5276 (3)	0.0281 (5)	
H27	0.391259	0.800913	0.567407	0.034*	
C27A	0.4246 (3)	0.8592 (2)	0.4045 (2)	0.0202 (4)	
Mn1	0.84578 (4)	0.19104 (3)	0.25082 (3)	0.02042 (9)	
Cl1	0.57864 (6)	0.18756 (6)	0.13323 (6)	0.02657 (13)	
Cl2	0.98756 (7)	0.30020 (6)	0.15037 (6)	0.02681 (13)	
Cl3	0.90155 (8)	−0.02287 (6)	0.24168 (7)	0.03208 (14)	
Cl4	0.89538 (7)	0.30780 (6)	0.47087 (6)	0.03045 (14)	

### Atomic displacement parameters (Å^2^)

**Table:**

	*U*^11^	*U*^22^	*U*^33^	*U*^12^	*U*^13^	*U*^23^
C11	0.0187 (10)	0.0201 (10)	0.0247 (10)	0.0026 (8)	0.0079 (8)	0.0028 (8)
N12	0.0148 (8)	0.0185 (8)	0.0206 (8)	0.0023 (6)	0.0062 (7)	0.0017 (7)
C12	0.0172 (10)	0.0271 (12)	0.0339 (12)	0.0024 (8)	0.0117 (9)	0.0042 (10)
C13	0.0160 (9)	0.0213 (10)	0.0218 (10)	0.0043 (8)	0.0049 (8)	0.0026 (8)
N13A	0.0225 (10)	0.0232 (10)	0.0312 (10)	0.0030 (8)	0.0089 (8)	−0.0013 (8)
C14	0.0178 (10)	0.0263 (11)	0.0294 (12)	0.0050 (8)	0.0081 (9)	−0.0011 (9)
C15	0.0176 (11)	0.0306 (13)	0.0439 (15)	−0.0008 (9)	0.0141 (10)	−0.0066 (11)
C16	0.0377 (15)	0.0282 (13)	0.0479 (16)	−0.0028 (11)	0.0280 (13)	0.0018 (12)
C17	0.0370 (14)	0.0231 (12)	0.0355 (13)	0.0020 (10)	0.0187 (11)	0.0067 (10)
C17A	0.0139 (9)	0.0119 (8)	0.0185 (9)	0.0009 (7)	0.0051 (7)	0.0005 (7)
C21	0.0221 (11)	0.0229 (11)	0.0291 (12)	0.0023 (9)	0.0108 (9)	−0.0015 (9)
N22	0.0305 (11)	0.0218 (10)	0.0236 (10)	0.0055 (8)	0.0063 (8)	0.0005 (8)
C22	0.0420 (16)	0.0320 (14)	0.0273 (13)	0.0125 (12)	0.0011 (11)	0.0036 (11)
C23	0.0343 (13)	0.0219 (11)	0.0236 (11)	0.0024 (9)	0.0138 (10)	0.0018 (9)
N23A	0.0243 (10)	0.0186 (9)	0.0274 (10)	−0.0005 (7)	0.0124 (8)	0.0001 (7)
C24	0.0201 (11)	0.0262 (12)	0.0385 (13)	−0.0014 (9)	0.0143 (10)	−0.0069 (10)
C25	0.0244 (12)	0.0270 (13)	0.0378 (14)	0.0041 (10)	0.0012 (10)	−0.0043 (11)
C26	0.0438 (16)	0.0223 (12)	0.0267 (12)	0.0001 (11)	0.0036 (11)	0.0054 (10)
C27	0.0356 (13)	0.0225 (11)	0.0275 (12)	−0.0050 (10)	0.0151 (10)	0.0010 (9)
C27A	0.0196 (10)	0.0180 (10)	0.0242 (10)	−0.0008 (8)	0.0114 (8)	−0.0003 (8)
Mn1	0.01803 (16)	0.02251 (18)	0.01970 (17)	0.00204 (12)	0.00538 (13)	0.00445 (13)
Cl1	0.0187 (2)	0.0328 (3)	0.0268 (3)	0.0030 (2)	0.0047 (2)	0.0100 (2)
Cl2	0.0266 (3)	0.0264 (3)	0.0307 (3)	0.0014 (2)	0.0136 (2)	0.0072 (2)
Cl3	0.0350 (3)	0.0268 (3)	0.0395 (3)	0.0103 (2)	0.0161 (3)	0.0133 (3)
Cl4	0.0269 (3)	0.0380 (3)	0.0222 (3)	−0.0009 (2)	0.0062 (2)	0.0003 (2)

### Geometric parameters (Å, º)

**Table:**

C11—N12	1.337 (3)	C21—H21	0.95
C11—C17A	1.347 (3)	N22—C23	1.332 (3)
C11—H11	0.95	N22—C22	1.468 (3)
N12—C13	1.366 (3)	C22—H22A	0.98
N12—C12	1.464 (3)	C22—H22B	0.98
C12—H12A	0.98	C22—H22C	0.98
C12—H12B	0.98	C23—N23A	1.346 (3)
C12—H12C	0.98	C23—H23	0.95
C13—N13A	1.363 (3)	N23A—C24	1.399 (3)
C13—H13	0.95	N23A—C27A	1.400 (3)
N13A—C17A	1.405 (3)	C24—C25	1.346 (4)
N13A—C14	1.414 (3)	C24—H24	0.95
C14—C15	1.350 (4)	C25—C26	1.414 (4)
C14—H14	0.95	C25—H25	0.95
C15—C16	1.425 (4)	C26—C27	1.343 (4)
C15—H15	0.95	C26—H26	0.95
C16—C17	1.349 (4)	C27—C27A	1.416 (3)
C16—H16	0.95	C27—H27	0.95
C17—C17A	1.400 (3)	Mn1—Cl3	2.3577 (7)
C17—H17	0.95	Mn1—Cl4	2.3674 (7)
C21—N22	1.366 (3)	Mn1—Cl1	2.3725 (7)
C21—C27A	1.369 (3)	Mn1—Cl2	2.3777 (7)
			
N12—C11—C17A	107.34 (19)	C23—N22—C21	110.4 (2)
N12—C11—H11	126.3	C23—N22—C22	124.2 (2)
C17A—C11—H11	126.3	C21—N22—C22	125.3 (2)
C11—N12—C13	110.61 (19)	N22—C22—H22A	109.5
C11—N12—C12	125.11 (19)	N22—C22—H22B	109.5
C13—N12—C12	124.3 (2)	H22A—C22—H22B	109.5
N12—C12—H12A	109.5	N22—C22—H22C	109.5
N12—C12—H12B	109.5	H22A—C22—H22C	109.5
H12A—C12—H12B	109.5	H22B—C22—H22C	109.5
N12—C12—H12C	109.5	N22—C23—N23A	107.7 (2)
H12A—C12—H12C	109.5	N22—C23—H23	126.2
H12B—C12—H12C	109.5	N23A—C23—H23	126.2
N13A—C13—N12	106.9 (2)	C23—N23A—C24	130.2 (2)
N13A—C13—H13	126.5	C23—N23A—C27A	108.6 (2)
N12—C13—H13	126.5	C24—N23A—C27A	121.2 (2)
C13—N13A—C17A	106.65 (18)	C25—C24—N23A	118.0 (2)
C13—N13A—C14	133.6 (2)	C25—C24—H24	121
C17A—N13A—C14	119.8 (2)	N23A—C24—H24	121
C15—C14—N13A	118.2 (2)	C24—C25—C26	121.7 (2)
C15—C14—H14	120.9	C24—C25—H25	119.1
N13A—C14—H14	120.9	C26—C25—H25	119.1
C14—C15—C16	121.5 (2)	C27—C26—C25	121.0 (2)
C14—C15—H15	119.2	C27—C26—H26	119.5
C16—C15—H15	119.2	C25—C26—H26	119.5
C17—C16—C15	121.1 (2)	C26—C27—C27A	118.9 (2)
C17—C16—H16	119.5	C26—C27—H27	120.5
C15—C16—H16	119.5	C27A—C27—H27	120.5
C16—C17—C17A	118.4 (2)	C21—C27A—N23A	106.4 (2)
C16—C17—H17	120.8	C21—C27A—C27	134.5 (2)
C17A—C17—H17	120.8	N23A—C27A—C27	119.0 (2)
C11—C17A—C17	130.5 (2)	Cl3—Mn1—Cl4	115.23 (3)
C11—C17A—N13A	108.49 (19)	Cl3—Mn1—Cl1	106.36 (3)
C17—C17A—N13A	121.0 (2)	Cl4—Mn1—Cl1	105.81 (3)
N22—C21—C27A	106.9 (2)	Cl3—Mn1—Cl2	110.21 (3)
N22—C21—H21	126.5	Cl4—Mn1—Cl2	107.51 (3)
C27A—C21—H21	126.5	Cl1—Mn1—Cl2	111.72 (2)
			
C17A—C11—N12—C13	−0.3 (3)	C27A—C21—N22—C23	0.2 (3)
C17A—C11—N12—C12	−178.7 (2)	C27A—C21—N22—C22	−178.2 (2)
C11—N12—C13—N13A	0.1 (2)	C21—N22—C23—N23A	−0.3 (3)
C12—N12—C13—N13A	178.5 (2)	C22—N22—C23—N23A	178.2 (2)
N12—C13—N13A—C17A	0.1 (2)	N22—C23—N23A—C24	−179.9 (2)
N12—C13—N13A—C14	−179.8 (2)	N22—C23—N23A—C27A	0.3 (3)
C13—N13A—C14—C15	−178.1 (2)	C23—N23A—C24—C25	−179.8 (2)
C17A—N13A—C14—C15	2.0 (3)	C27A—N23A—C24—C25	0.0 (3)
N13A—C14—C15—C16	−0.7 (4)	N23A—C24—C25—C26	−1.6 (4)
C14—C15—C16—C17	−1.1 (4)	C24—C25—C26—C27	2.2 (4)
C15—C16—C17—C17A	1.3 (4)	C25—C26—C27—C27A	−1.1 (4)
N12—C11—C17A—C17	−178.0 (2)	N22—C21—C27A—N23A	0.0 (2)
N12—C11—C17A—N13A	0.4 (2)	N22—C21—C27A—C27	178.7 (3)
C16—C17—C17A—C11	178.4 (2)	C23—N23A—C27A—C21	−0.1 (3)
C16—C17—C17A—N13A	0.1 (4)	C24—N23A—C27A—C21	180.0 (2)
C13—N13A—C17A—C11	−0.3 (2)	C23—N23A—C27A—C27	−179.1 (2)
C14—N13A—C17A—C11	179.6 (2)	C24—N23A—C27A—C27	1.0 (3)
C13—N13A—C17A—C17	178.3 (2)	C26—C27—C27A—C21	−179.1 (3)
C14—N13A—C17A—C17	−1.8 (3)	C26—C27—C27A—N23A	−0.5 (3)

### Hydrogen-bond geometry (Å, º)

**Table:**

*D*—H···*A*	*D*—H	H···*A*	*D*···*A*	*D*—H···*A*
C11—H11···Cl4	0.95	2.76	3.592 (2)	146
C13—H13···Cl1^i^	0.95	2.66	3.422 (2)	138
C14—H14···Cl1^i^	0.95	2.79	3.549 (3)	137
C21—H21···Cl3^ii^	0.95	2.80	3.632 (2)	147
C22—H22*C*···Cl2^i^	0.98	2.82	3.742 (3)	156
C23—H23···Cl1^iii^	0.95	2.81	3.440 (3)	125
C24—H24···Cl3^iii^	0.95	2.69	3.573 (3)	154
C27—H27···Cl4^iv^	0.95	2.77	3.600 (3)	146

Symmetry codes: (i) −*x*+1, −*y*+1, −*z*; (ii) *x*−1, *y*+1, *z*; (iii) *x*, *y*+1, *z*; (iv) −*x*+1, −*y*+1, −*z*+1.
